# Alcohol consumption in relation to cardiovascular diseases and mortality: a systematic review of Mendelian randomization studies

**DOI:** 10.1007/s10654-021-00799-5

**Published:** 2021-08-22

**Authors:** Inge A. T. van de Luitgaarden, Sabine van Oort, Emma J. Bouman, Linda J. Schoonmade, Ilse C. Schrieks, Diederick E. Grobbee, Yvonne T. van der Schouw, Susanna C. Larsson, Stephen Burgess, Adriana J. van Ballegooijen, N. Charlotte Onland-Moret, Joline W. J. Beulens

**Affiliations:** 1 UMC Utrecht, Julius Center for Health Sciences and Primary Care, Utrecht University, Utrecht, The Netherlands; 2 Amsterdam UMC locatie VUmc, Department of Epidemiology and Data Science, Amsterdam Cardiovascular Sciences Research Institute and Amsterdam Public Health Research Institute, Vrije Universiteit Amsterdam, Amsterdam, The Netherlands; 3 Vrije Universiteit Amsterdam, University Library, Amsterdam, The Netherlands; 4 Julius Clinical, Zeist, The Netherlands; 5 Department of Surgical Sciences, Uppsala University, Uppsala, Sweden; 6 Unit of Cardiovascular and Nutritional Epidemiology, Institute of Environmental Medicine, Karolinska Institutet, Stockholm, Sweden; 7 MRC Biostatistics Unit, University of Cambridge, Cambridge, UK; 8 Department of Public Health and Primary Care, University of Cambridge, Cambridge, UK; 9 Department of Nephrology, Amsterdam UMC, Vrije Universiteit Amsterdam, Amsterdam, The Netherlands

**Keywords:** Systematic review, Mendelian randomization, Alcohol consumption, Cardiovascular disease, Diabetes, Cardiovascular risk factors, Mortality

## Abstract

The causal effects of alcohol-in-moderation on cardiometabolic health are continuously debated. Mendelian randomization (MR) is an established method to address causal questions in observational studies. We performed a systematic review of the current evidence from MR studies on the association between alcohol consumption and cardiometabolic diseases, all-cause mortality and cardiovascular risk factors. We performed a systematic search of the literature, including search terms on type of design and exposure. We assessed methodological quality based on key elements of the MR design: use of a full instrumental variable analysis and validation of the three key MR assumptions. We additionally looked at exploration of non-linearity. We reported the direction of the studied associations. Our search yielded 24 studies that were eligible for inclusion. A full instrumental variable analysis was performed in 17 studies (71%) and 13 out of 24 studies (54%) validated all three key assumptions. Five studies (21%) assessed potential non-linearity. In general, null associations were reported for genetically predicted alcohol consumption with the primary outcomes cardiovascular disease (67%) and diabetes (75%), while the only study on all-cause mortality reported a detrimental association. Considering the heterogeneity in methodological quality of the included MR studies, it is not yet possible to draw conclusions on the causal role of moderate alcohol consumption on cardiometabolic health. As MR is a rapidly evolving field, we expect that future MR studies, especially with recent developments regarding instrument selection and non-linearity methodology, will further substantiate this discussion.

**Supplementary Information:**

The online version contains supplementary material available at 10.1007/s10654-021-00799-5.

## Introduction

The alleged beneficial effects of moderate consumption on cardiometabolic health and mortality in comparison to abstainers and heavy drinkers have been discussed for decades [1, 2]. This potentially non-linear, or J-shaped, relationship has been consistently shown in observational studies for cardiovascular mortality and certain cardiometabolic diseases including myocardial infarction and diabetes [3–5]. These findings are debated as they may be biased by including former drinkers in the abstainer reference group [6], and also through residual confounding and reverse causation [7]. Randomized intervention studies by design do not suffer from these types of biases. Thus far, mainly short-term randomized controlled trials with cardiometabolic biomarkers as endpoints have been carried out [8–13]. According to several meta-analyses of these trials moderate alcohol intake increased HDL cholesterol and adiponectin, and lowered fasting insulin and HbA1c levels, but had no effect on triglycerides and insulin sensitivity [8, 9, 11]. The literature reports a dose–response relation between alcohol consumption and blood pressure, which is particularly apparent for heavy drinkers [10, 12, 13]. Another trial showed beneficial effects of introducing alcohol abstinence in regular drinkers with atrial fibrillation on arrhythmias [14]. However, a long-term, randomized clinical trial (RCT) with clinical endpoints would provide the best evidence to draw conclusions on causality, but is expensive, time-consuming and even the conduct of such a trial is a source of debate itself [15–18].

**Fig. 1:**
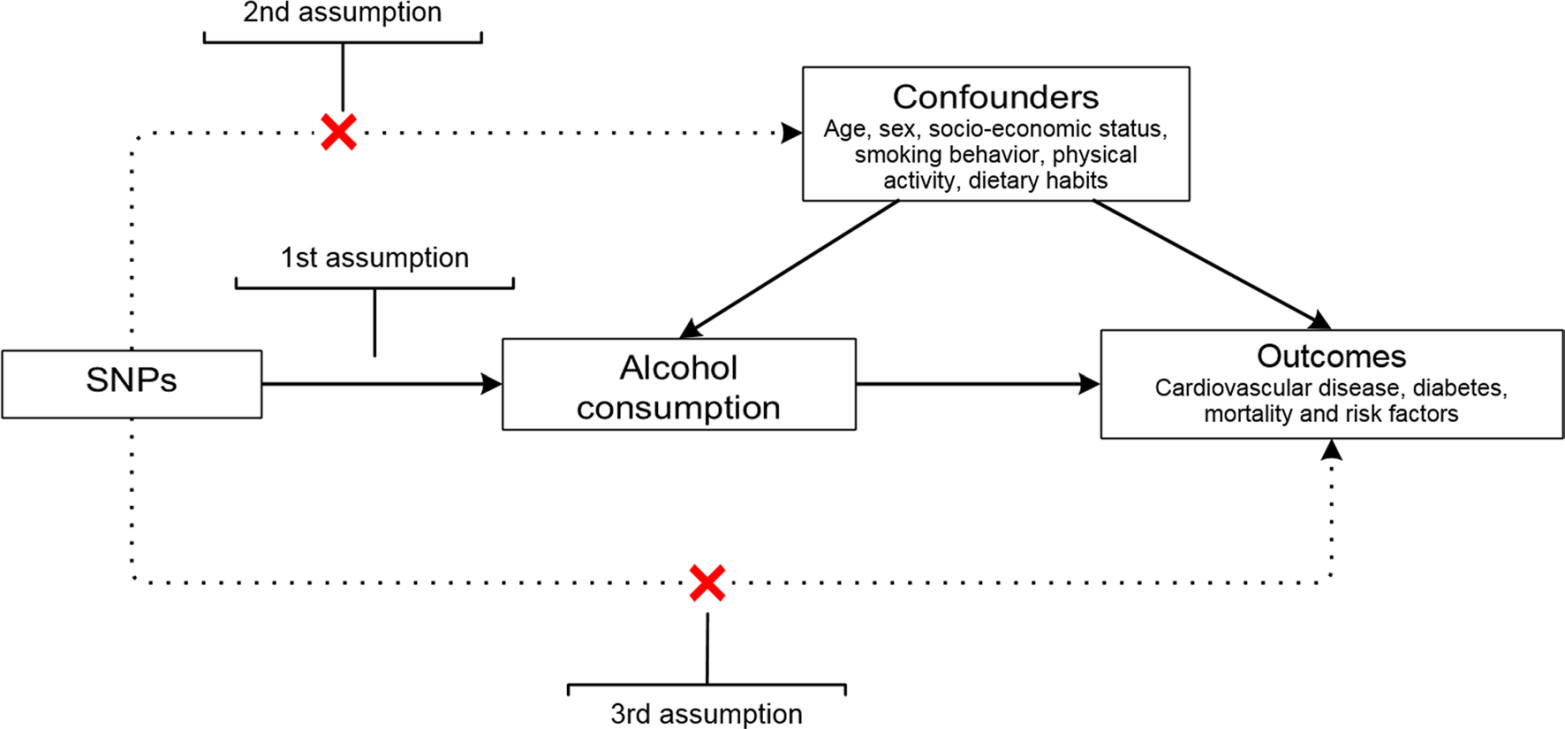
Overview of the Mendelian randomization design and assumptions. First assumption: the genetic variant is associated with alcohol consumption. Second assumption: the genetic variant is not associated with any confounder of the alcohol consumption-outcome association. Third assumption: the genetic variant does not affect the outcome, except possibly via its association with alcohol consumption

Recently, the Mendelian randomization (MR) approach has gained popularity for studying causal effects in observational research by using genetic variants that fulfill instrumental variable (IV) assumptions. The MR approach is a type of IV analysis, in which genetic variants are used as proxies for exposure status (Fig. 1) [19]. Unlike the risk factor of interest, genetic variants are randomly allocated at conception and therefore not related to potential confounders. As such, this type of observational study design mimics the features of a randomized trial and could potentially be a method to study alcohol consumption without the aforementioned problems, provided that all assumptions related to the MR design hold. Another important advantage of MR is that it is thought to reflect the lifetime exposure of a certain risk factor [20].

Because the instrument serves as proxy for exposure status, it is essential that this instrument is valid. To ensure validity, three key assumptions need to be met: the genetic variant (1) is robustly associated with the exposure, (2) is not associated with any confounder of the exposure-outcome association, and (3) only affects the outcome via its association with the exposure (Fig. 1). The first studies that used genetic variants to investigate the association between alcohol and cardiometabolic outcomes have focused on variation in the genes that are known to play a role in alcohol metabolism: *ALDH2* and *ADH1B/C*. Functional variants in these genes lead to accumulation of the toxic degradation product acetaldehyde, which is associated with adverse effects (e.g. flushing, nausea) and, consequently, with limited alcohol consumption in people carrying them [21]. These variants can therefore be suitable instruments and mainly the *ALDH2* variant explains a quite high proportion of the variance in alcohol consumption in Asian populations. However, for European populations the functional variants explain only a small part of the variance in alcohol consumption and therefore analyses may have low power. Large genome-wide association studies (GWAS) have discovered more variants statistically significantly associated with alcohol consumption, without necessarily being causal variants, but often fail to detect the infrequent functional variants. This has led to better-powered MR analyses, but also to less straightforward validation of the assumptions.

Another challenge in the MR design is the assessment of potential non-linear relationships, which is of particular interest when studying alcohol and cardiometabolic outcomes. MR studies could thus help to elucidate the causal relation of alcohol consumption with cardiovascular diseases, but an overview of the evidence and quality of these studies is lacking.

With this systematic review, we aimed to assess the methodological quality and provide an overview of the current evidence from MR studies on the causal relationship between alcohol consumption, mortality, cardiometabolic diseases and risk factors for cardiometabolic disease.

## Methods

### Search strategy

This systematic review was conducted in accordance with the Preferred Reporting Items for Systematic Reviews and Meta-Analysis (PRISMA) statement [22].

A comprehensive literature search was performed to identify all MR studies that used a genetic instrument as proxy for alcohol exposure in relation to any cardiometabolic disease, all-cause mortality, or cardiometabolic risk factors. PubMed, Embase and Scopus were searched from inception until November 16th, 2020, in collaboration with a medical librarian (L.S.). The search strategy included terms describing the exposure (“alcohol consumption”) and the study design (“Mendelian Randomization”, “instrumental variable analysis”). The full search strategy has been included in Supplementary Methods 1. Reference lists were manually checked to further identify potentially eligible studies. With this search strategy we did not aim to identify studies that assessed the association between a single genetic variant and one of our outcomes, but were not called MR study, because these studies were not set up as an MR study or instrumental variable analysis and our main goal was to provide an update on the current status and quality of MR research specifically. The protocol for the systematic review was registered with the International Prospective Register of Systematic Reviews (PROSPERO) on April 28th, 2020 (CRD42020151510).

### Study selection procedure

Two reviewers (S.O. and either E.B. or I.L.) independently reviewed each title and abstract, and, subsequently, full text. In case of discrepancies, a third researcher (I.L. or A.B.) was consulted to decide on in- or exclusion of the study. Studies were eligible for inclusion if meeting all of the following criteria: (1) the MR design was used, (2) alcohol consumption was assessed as exposure, and (3) cardiometabolic diseases, mortality, or cardiometabolic risk factors were used as outcome. We additionally excluded studies that (1) were non-human studies, (2) were written in another language than English or Dutch, (3) were conference abstracts, reviews or editorials, or (4) had no full-text available.

### Data extraction

The primary outcomes of this systematic review were: (1) cardiovascular diseases (including stroke, myocardial infarction, coronary artery disease, heart failure, atrial fibrillation, and peripheral artery disease); (2) type 2 diabetes mellitus and non-alcoholic fatty liver disease; and (3) all-cause and cause-specific mortality. Our secondary outcomes were risk factors for cardiometabolic diseases, including: (1) anthropometric measures (body mass index (BMI), waist circumference, waist-to-hip-ratio, overweight or obesity); (2) blood pressure (systolic and diastolic blood pressure and hypertension); (3) lipids (total cholesterol (total-C), HDL cholesterol (HDL-C), LDL cholesterol (LDL-C), and triglycerides (TG)) and (4) glucose-related risk factors (HbA1c, adiponectin level, fasting glucose, insulin sensitivity, and insulin resistance). We included classical cardiometabolic risk factors as secondary outcomes in this review, because these are thought to be intermediates on the path to CVD. However, some of these mechanisms may still be uncertain, as for example the cardioprotective effects of HDL-C are currently subject to debate [23].

We used a comprehensive questionnaire published by Grover et al. as guidance for data extraction [24]. We made the distinction between one and two-sample MR designs: one-sample studies are performed in a single study population, whereas two-sample studies combine summary statistics on the gene-exposure association and the gene-outcome association from different data sources. Per study two reviewers (I.L and either S.O. or J.B.) extracted, independent from each other, the following data: first author’s name, year of publication, design of the study (one-sample or two-sample MR), data source(s), sample size, ancestry, sex and age distribution (in one-sample MR studies only), genetic instrument, assessed outcomes, the effect estimates with confidence intervals, and information for the methodological quality assessment.

### Methodological quality assessment

Currently, there is no quality assessment tool available for systematic reviews on MR studies. We therefore rated the methodological quality of the included studies based on criteria that are key elements of the MR design: whether and what type of IV analysis was performed and whether the three MR assumptions were checked and not violated. In addition, we checked whether potential non-linearity was addressed and added that to the quality assessment. The methodological quality of the included studies was independently assessed by two reviewers (I.L and either S.O. or J.B.). Any inconsistencies were resolved by discussion until consensus was reached.

The first element of the MR design is the use of a full IV analysis, which is important to be able to estimate the size of the causal effect [19]. Common statistical methods for a full IV analysis are two-stage least squares regression (2SLS), ratio of coefficients and generalized method of moments (GMM) for the one-sample MR design, and the inverse-variance weighted method (IVW) for the two-sample MR design. Sometimes, MR studies do not perform a full IV analysis, but use a different approach, such as an association analysis between the genetic variant and outcome in which the number of allele copies is used as level of exposure. Although this method is sufficient to investigate whether the association is causal, it cannot quantify the size of the causal effect. A method that is used less often is comparison of the observed genotype-outcome associations with the genotype-outcome associations that are expected if the exposure-outcome association were truly causal [25]. This method is also less suitable to quantify causal effect sizes [25]. If a full IV analysis was performed, we rated this element as “good”, if a different method was applied, we rated this element as “poor”.

#### Validation of the assumptions

In IV analysis—and thus in MR analysis—the validity of the instrument is essential. In order for a genetic instrument to be valid, the three key assumptions mentioned in the introduction need to be met (Fig. 1) [19].

Validation of the first assumption in one-sample MR studies is typically evaluated by regressing the genetic instrument on the exposure to test the strength of the association. Traditionally, the F-statistic of this association is provided, in which an F-statistic > 10 is regarded sufficient to overcome weak instrument bias [26]. In the two-sample MR design, validation of the first assumption is assured by selecting only SNPs that are strongly (genome-wide significant) and robustly (replicated in another independent sample) associated with the exposure. These GWAS often report the phenotypical variance explained (r^2^) of all genome-wide significant SNPs combined. If the first assumption was validated by either testing and providing an F-statistic (one-sample MR studies) or by selecting strongly and robustly associated SNPs from GWAS (two-sample MR studies), we rated this element as “good”. We rated it “moderate” if the first assumption was verified in a different way, and “poor” if validation of the first assumption was not done or reported.

Because genetic variants are randomly allocated at conception, they are assumed not to be associated with potential confounding factors. To verify whether this second assumption holds, associations between the genetic instrument and confounding factors can be tested and reported.

The last assumption, also known as the exclusion-restriction assumption, could be violated if the genetic instrument affected the outcome through factors other than the exposure of interest. This is also called (horizontal) pleiotropy. Although it is impossible to prove that this assumption holds, its plausibility can be checked, for example by using a control group that is not exposed to the factor of interest (negative controls or no-relevance group), this case the abstainers. If the genetic instrument would only exert its effect through the exposure, then absence of that exposure would automatically lead to a null association between genetic instrument and outcome. For alcohol consumption as exposure, non-drinkers would make an obvious control group. Likewise, in some cultures women tend to abstain from alcohol and therefore could be valid negative controls as well. Sometimes, the validity of the third assumption can be assumed when the biological function of the genetic instrument is known. For both the second and third assumption, SNPs that are suspected to have pleiotropic effects can be excluded in the selection process. More formal techniques to assess potential pleiotropy include the use of MR-Egger regression, and relatively new techniques such as MR-PRESSO (Mendelian Randomization Pleiotropy RESidual Sum and Outlier) [27]. Validation of both the second and third assumption was rated “good” if these assumptions were tested, “moderate” if validity was assumed based on literature, and “poor” if validation of these assumptions was not reported.

#### Non-linearity

Since observational studies suggest that the association between alcohol consumption and cardiometabolic outcomes and mortality (i.e., our primary outcomes) is J- or U-shaped (i.e., non-linear), we assessed whether the MR studies explored potential non-linearity in their analysis. Recently, the use of localized average causal effects (LACEs) has been proposed as a technique to assess non-linearity in a one-sample MR setting [28, 29]. If studies reported exploration of non-linearity, we rated this element as “good”. If no non-linear analyses were performed, we rated this as “poor”.

## Results

**Fig. 2:**
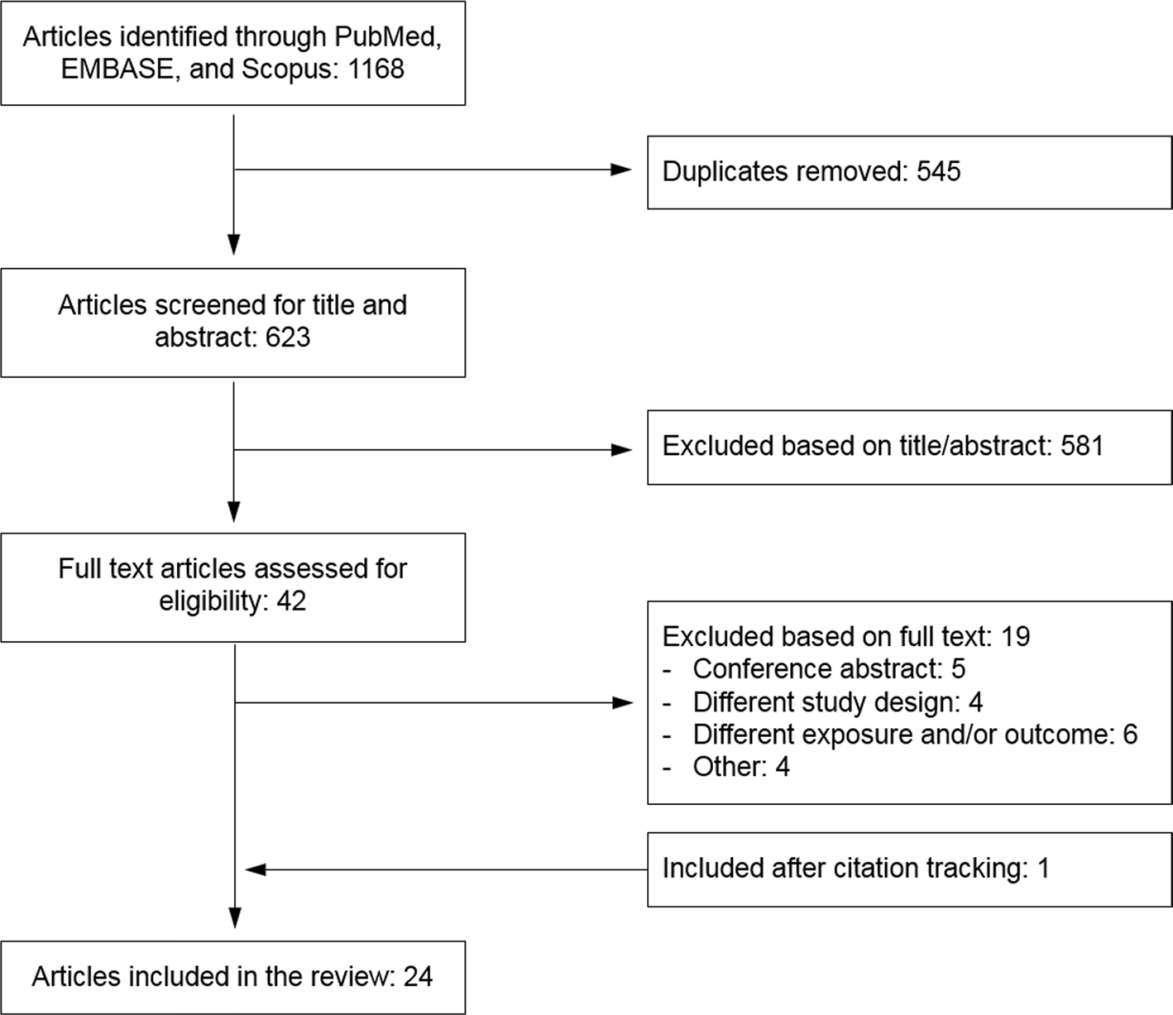
Flowchart of the selection of Mendelian randomization studies of alcohol consumption in relation to cardiovascular diseases, diabetes, mortality or cardiometabolic risk factors

The search strategy resulted in 1168 studies, of which 545 duplicates were removed (Fig. 2). After title/abstract screening another 581 studies were excluded, with the main reasons for exclusion being: use of a study design other than MR (N = 279), different study exposure or outcome (N = 181) and non-human studies (N = 104). After full-text screening, 23 studies were included in the current systematic review [30–52]. Citation tracking resulted in one additional publication [53]. The 24 included studies were published between 2008 and 2020.

Eighteen studies (75%) used data from a single study population (one-sample MR), with one study being a meta-analysis applying the one-sample MR approach [32], while the six most recent studies used the two-sample MR design (Table 1). The included studies were either performed in Asian populations (50%) or populations of European ancestry (50%). Over half of the included studies used a single, functional SNP as genetic instrument (58%), which was either rs671 (located in the *ALDH2* gene region) in populations of Asian ancestry (79% of the studies using a single SNP) or rs1229984 (in *ADH1B*) in populations of European ancestry (21%). Since the *ALDH2*-rs671 SNP is monomorphic (i.e., only one allele exists) in populations of European ancestry, this genetic variant cannot be used as an IV for alcohol consumption in European studies [43]. Two studies used a combination of *ADH1B*-rs1229984 and *ADH1C*-rs698, and one study combined *ADH1B*-rs1229984 with *ALDH2*-rs671. Seven studies performed the IV analysis with a genetic risk score or a combination of several SNPs as instrument, ranging from 5 to 94 included SNPs. Selection of the genetic instrument in the majority of studies (71%) was based on the biological function of the genetic variant. However, in the two-sample MR studies and studies using a genetic risk score as instrument, SNPs were selected from a published GWAS [47]. Thirteen studies (54%) assessed one or more of the primary outcomes (cardiovascular disease, diabetes, or mortality), and the other eleven studies assessed cardiometabolic risk factors only.

**Table 1:** Overview of the 24 included Mendelian randomization studies on alcohol consumption in relation to cardiovascular diseases, diabetes, mortality or cardiometabolic risk factors, stratified by one-sample and two-sample study design

Ancestry	N SNPs	SNP	Study	Sample size	Male sex	Age*	Country	Outcomes			
								CVD	Diabetes	Mortality	Risk factors
*One sample MR studies (N* = *18)*											
Asian	1	ALDH2 (rs671)	AuYeung^a^ [30]	4867	100%	≥ 50	China	CVD, IHD			BMI, SBP, DBP, HDL-C, LDL-C, triglycerides, FG
			AuYeung^a^ [31]	4867	100%	≥ 50	China	CVD, IHD			SBP, DBP, HDL-C, LDL-C, triglycerides, FG
			Chen^b^ [32]	7658 (BP) 4219 (HT)	Mixed	NA	Japan				SBP, DBP, HT
			Cho^c^ [33]	7152	47%	52 ± 8	South Korea	CVD, CHD	DM		BMI, WC, WHR, SBP, DBP, HT, TC, HDL-C, LDL-C, TG, FG
			Jee [34]	4367	69%	M: 42 ± 9 F: 43 ± 11	South Korea				FG
			Peng [35]	4536	50%	55 ± 7	China		DM		BMI, WC, WHR, SBP, DBP, TC, HDL-C, TG, FG; HOMA-IR; HOMA-beta; PPG
			Tabara [36]	4229	54%	63 ± 11	Japan				HDL-C, LDL-C
			Tabara [37]	8,364	33%	M: 55 ± 14 F: 51 ± 13	Japan				HDL-C, LDL-C, TG
			Taylor [38]	3788	36%	58 ± 11	China				BMI, SBP, DBP, TC, HDL-C, LDL-C, TG, FG
			Zhao [39]	2349	46%	59 ± 11	China				BMI, weight, WC, SBP, DBP, HT, TC, HDL-C, LDL-C, TG
			Cho^cd^ [40]	2011	67%	56 ± 7	South Korea				SBP, DBP, HT
	2	ALDH2 (rs671), ADH1B (rs1229984)									
			Millwood [41]	161,498	41%	52 ± 11	China	AMI, CHD, stroke			BMI, weight, WC, WHR, SBP, DBP, HDL-C, LDL-C, TG, NFG
European	1	ADH1B (rs1229984)	Almeida [42]	3496	100%	77 ± 4	Australia			All-cause	
			Holmes^e^ [43]	261,991	52%	58 (26; 75)	Europe and North America	CHD, stroke	T2DM		BMI, WC, SBP, DBP, HT, HDL-C, TG, FG
			Silverwood^e^ [46]	80,057	NA	NA	Europe and North America				BMI, WC, SBP, HDL-C, TG
	2	ADH1B (rs1229984), ADH1C (rs698)	Christensen [44]	74,632	45%	57 (20; 99)	Denmark	Stroke			
			Lawlor [45]	54,604	43%	56 ± 13	Denmark				BMI, SBP, DBP, HDL-C, TG, NFG
	5	ADH1B (rs2066702 and rs1693457), ADH1B/1C (rs1789891), ADH1C (rs698), ADH4 (rs1126671)	Vu^e^ [47]	10,893	47%	54 ± 6	United States (European Americans)				TC, HDL-C, LDL-C, TG

Ancestry	Data source exposure	N SNPs	Study	Sample size outcome	Data source outcomes	Outcomes			
						CVD	Diabetes	Mortality	Risk factors
*Two-sample MR studies (N* = *6)*									
European	GSCAN	43	Jiang [48]	60,620 cases, 970,216 controls	Meta-analysis of 6 studies/consortia: HUNT, deCODE, MGI, DiscovEHR, UKB and the AFGen Consortium	AF			
		94	Larsson [49]	Between 184,305 and 588,190 per outcome	Different data sources for different outcomes: MEGASTROKE Consortium, UKB, ISGC, (CARDIoGRAMplusC4D) consortium, AFGen Consortium	stroke, CHD, AF, HF, PAD			SBP, DBP, HDL-C, LDL-C, TG
		91	Van Oort [50]	47,309 cases, 930,014 controls	Heart Failure Molecular Epidemiology for Therapeutic Targets Consortium (26 studies)	HF			
		85	Van Oort [51]	UKB:54,358 cases, 408,652 controls FG: 15,870 cases, 74,345 controls	UKB; FinnGen consortium				HT
		89	Van Oort [53]	11,262 cases, 25,483 controls	Meta-analysis of 20 European studies on longevity			Longevity	
		83	Yuan [52]	74,124 cases, 824,006 controls	DIAGRAM consortium (32 studies); FinnGen consortium as replication cohort		DM		

^*^Age (years) displayed as mean ± SD, mean (min; max), or > min

^a^Same study population

^b^Meta-analysis applying the instrumental variable approach

^c^Overlapping study population

^d^Main analysis with only rs671 as genetic instrument; sensitivity analysis with both SNPs as genetic instrument

^e^Overlapping study population

*AMI* acute myocardial infarction, *AF* atrial fibrillation, *BMI* body mass index, *CHD* coronary heart disease, *CVD* cardiovascular disease, *DBP* diastolic blood pressure, *DM* diabetes mellitus, *F* women, *FG* fasting glucose, *GSCAN* GWAS & sequencing consortium of alcohol and nicotine use, *HDL-C* high-density lipoprotein cholesterol, *HF* heart failure, *HOMA-IR* homeostatic model assessment of insulin resistance, *HOMA-beta* homeostatic model assessment of beta-cell function, *HT* hypertension, *IHD* ischemic heart disease, *M* men, *NA* not available, *NFG* non-fasting glucose, *PAD* peripheral artery disease, *PPG* 2-h post-prandial blood glucose, *SBP* systolic blood pressure, *SNP* single nucleotide polymorphism, *T2DM* type 2 diabetes mellitus, *TC* total cholesterol, *TG* triglycerides, *UKB* UK Biobank, *WC* waist circumference, *WHR* waist-to-hip ratio

### Methodological quality assessment

**Fig. 3:**
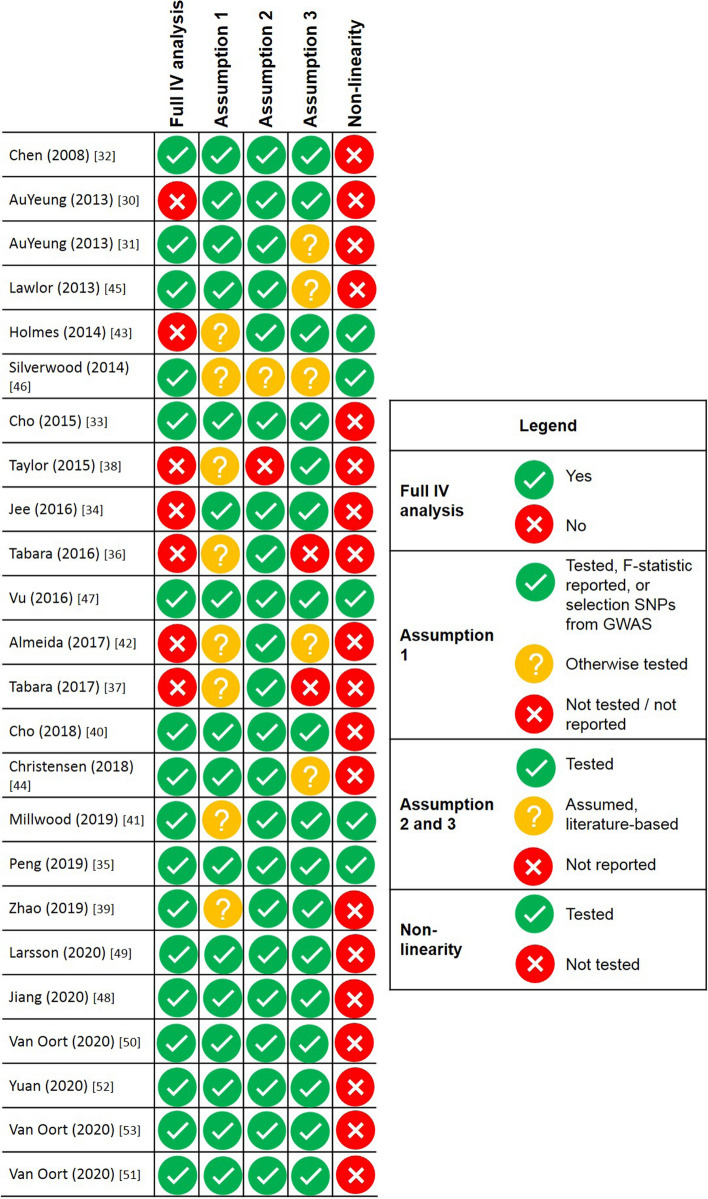
Methodological quality assessment of the included Mendelian randomization studies, sorted by year of publication and first author name. Please see Fig. 1 for an overview of the assumptions of a Mendelian randomization analysis. First assumption: the genetic variant is associated with alcohol consumption. Second assumption: the genetic variant is not associated with any confounder of the alcohol consumption-outcome association. Third assumption: the genetic variant does not affect the outcome, except possibly via its association with alcohol consumption. Please see Supplementary Table 1 for a more extensive overview of the methodological quality assessment

The assessment of methodological quality of the studies included in this review has been provided in Fig. 3 and Supplementary Table 1. Seventeen of the 24 included studies (71%) performed a full IV analysis with 2SLS regression (in one-sample MR) and IVW (in two-sample MR) as the most common methods used for IV analysis.

Half of the studies verified all three key assumptions. All studies tested the first assumption. All six two-sample MR studies referred to the SNP selection in the GWAS as means of validation. However, only ten of the 18 (59%) one-sample MR studies reported an F-statistic. The reported F-statistics of these ten studies were all > 10, suggesting that the instruments used were sufficiently strong in these analyses. Regarding the second assumption, most studies (92%) reported to have assessed the association between the instrument and potential confounders and for one study [46] we assumed that this assumption was tested as it was a continuation of a previously reported analysis [43]. Twenty-two studies (92%) validated the third assumption in their publication, of which ten studies used negative controls, five studies assumed that pleiotropy was not present based on the literature or previous analyses, one study controlled for pleiotropy by excluding SNPs that were in linkage disequilibrium with outcome-related loci and six studies used formal statistical techniques such as MR-Egger and MR-PRESSO.

Five studies (21%) performed non-linear analyses, three for primary outcomes and two for secondary outcomes only. Three studies categorized self-reported alcohol consumption and tested the associations over the different categories [41, 43, 47] and two studies used the LACEs method [35, 46].

### Associations of genetically predicted alcohol consumption with mortality, cardiometabolic diseases and risk factors

Six out of the nine studies (67%) that assessed cardiovascular disease as outcome reported null associations. Similarly, null associations were found in 75% of the studies with diabetes as outcome. The study that assessed the linear association with all-cause mortality as outcome reported a detrimental association, whereas the study on longevity reported null associations only (Table 2).

**Table 2:** Overview of the associations of higher genetically predicted alcohol consumption with cardiovascular disease, diabetes and mortality in Mendelian randomization studies

Outcome and study	Ancestry	IV	Effect measure and unit	Association with outcome		
				Total	Men	Women
*Cardiovascular disease*						
AuYeung* [30]	Asian	No	OR GA versus AA genotype		1.14 (0.73; 1.79)	
AuYeung* [31]	Asian	Yes	OR per 10 g/day		0.98 (0.76; 1.27)	
Cho [33]	Asian	Yes	OR per g/day	0.95 (0.88; 1.03)	0.99 (0.97; 1.01)	1.21 (0.83; 1.76)
*Acute myocardial infarction*						
Millwood [41]	Asian	Yes	RR per 280 g/week		0.96 (0.78; 1.18)	0.94 (0.74; 1.20)
Ischemic heart disease/coronary heart disease						
AuYeung* [30]	Asian	No	OR GA versus AA genotype		1.48 (0.84; 2.61)	
AuYeung* [31]	Asian	Yes	OR per 10 g/day		1.10 (0.83; 1.45)	
Cho [33]	Asian	Yes	OR per g/day	0.98 (0.89; 1.09)	0.99 (0.97; 1.02)	1.00 (0.61; 1.62)
Holmes [43]	European	No	OR GG versus AA or AG	**1**.**11** (**1**.**04**; **1**.**19**)		
Larsson [49]	European	Yes	OR per 1-SD increase in log-transformed drinks/week	1.16 (1.00; 1.36)		
Millwood [41]	Asian	Yes	RR per 280 g/week		1.05 (0.94; 1.17)	1.02 (0.93; 1.12)
*Atrial fibrillation*						
Jiang^a^ [48]	European	Yes	OR per 1-SD increase in drinks/week	1.00 (0.77; 1.32)		
Larsson^a^ [49]	European	Yes	OR per 1-SD increase in log-transformed drinks/week	1.17 (1.00; 1.37)		
*Peripheral artery disease*						
Larsson [49]	European	Yes	OR per 1-SD increase in log-transformed drinks/week	**3**.**05** (**1**.**92**; **4**.**85**)		
*Heart failure*						
Larsson^b^ [49]	European	Yes	OR per 1-SD increase in log-transformed drinks/week	1.00 (0.68; 1.47)		
Van Oort^b^ [50]	European	Yes	OR per 1-SD increase in log-transformed drinks/week	1.11 (0.85; 1.46)		
*Stroke*						
Christensen [44]	European	Yes	HR slow versus fast metabolizers	1.15 (0.66; 2.02)		
Holmes [43]	European	No	OR GG versus AA or AG	1.02 (0.93; 1.11)		
Millwood [41]	Asian	Yes	RR per 280 g/week		**1**.**38** (**1**.**26**; **1**.**51**)	0.98 (0.88; 1.09)
Larsson [49]	European	Yes	OR per 1-SD increase in log-transformed drinks/week	**1**.**27** (**1**.**12**; **1**.**45**)		
*Diabetes*						
Cho [33]	Asian	Yes	OR per g/day	1.05 (0.99; 1.10)	1.02 (1.00; 1.04)	0.97 (0.77; 1.22)
Holmes [43]	European	No	OR GG versus AA or AG	0.98 (0.92; 1.05)		
Peng [35]	Asian	Yes	Incidence rate ratio for 22 g increase in log-transformed alcohol		**1**.**13** (**1**.**06**; **1**.**67**)	1.40 (0.67; 2.93)
Yuan [52]	European	Yes	OR per drinks/week	1.08 (0.80; 1.45)		
*All-cause mortality*						
Almeida [42]	European	No	HR non-carriers versus A-allele carriers		**1**.**47** (**1**.**15**; **1**.**85**)	
*Longevity*						
Van Oort [53]	European	Yes	OR per 1-SD increase in log-transformed drinks/week	0.87 (0.55; 1.38)		

*Same study population

^a^These two studies for atrial fibrillation had overlapping study populations

^b^These two studies for heart failure had overlapping study populations

The results presented here are the results of the linear analyses. Detrimental associations have been displayed in bold

*IV* instrumental variable analysis

The associations dispalyed bold are detrimental associations

For secondary outcomes, alcohol consumption was observed to be detrimental for the outcomes that included anthropometric (i.e., BMI, weight, weight circumference and waist-to-hip ratio) (Supplementary Table 2) and blood pressure measures (i.e., hypertension, systolic and diastolic blood pressure) (Supplementary Table 3). In contrast, alcohol consumption had beneficial associations with HDL-C and LDL-C, but were inconsistently associated with triglycerides (Supplementary Table 4). MR studies of the association of alcohol consumption with glycemic traits were relatively limited but generally reported detrimental associations in populations of Asian ancestry (Supplementary Table 5).

From the five studies investigating non-linearity, two studies found a non-linear trend for predicted alcohol consumption and several lipids, indicating that low-to-moderate alcohol consumers had a more favorable lipid profile compared to never drinkers [46, 47].The other three studies reported linear trends and did not find evidence for non-linearity. It is important to remark that there is a possibility that the studies investigating non-linearity by stratifying on alcohol consumption categories [41, 43, 47], introduced collider bias: conditioning on the exposure X, which is on the path between genetic instrument G and outcome Y, might induce an association between G and Y.

## Discussion

In the recent years, 24 studies using the MR design assessed the causal relation between alcohol consumption and mortality, cardiometabolic diseases or risk factors. Seventeen studies (71%) performed a full IV analysis, thirteen of them (54%) reported validation of all key assumptions and five (21%) explored potential non-linear associations. The majority of studies reported null associations for genetically predicted alcohol consumption with cardiometabolic diseases and mortality. In general, alcohol consumption was found to be detrimental for blood pressure, glucose, triglycerides and anthropometric measures including BMI, except for HDL-C and LDL-C, for which generally protective associations were reported. For most outcomes, similar associations were found regardless of the genetic variant used as IV (i.e., ALDH2, ADH1B or multiple SNPs). However, for HDL-C, the studies that used either ALDH2 or multiple SNPs as an IV reported positive associations with HDL-C, whereas studies that used ADH1B reported null associations. Moreover, the one study that looked at ALDH2 and ADH1B separately reported a much weaker association when ADH1B was used as IV as compared to ALDH2 [41]. Previous work has suggested pleiotropy or linkage disequilibrium of ADH1B with a variant related to HDL-C as potential explanation [54]. The discordance in results for triglyceride levels (i.e., most Asian studies report either positive or null associations, whereas most European studies report inverse associations) might potentially originate from the same bias. This emphasizes the importance of applying sensitivity analyses that account for or detect potential IV invalidation, as well as the need for multiple instruments [54].

The studies investigating non-linearity provided inconsistent results on the shape of the associations. When comparing the results of the MR studies in our systematic review with the non-linear associations which are often found in the observational literature [3–5], we can thus not provide a single clear answer on the definite shape of the associations for moderate drinkers. However, for excessive amounts of alcohol the evidence points consistently towards a harmful effect of alcohol on most cardiometabolic risk factors. The positive effect of alcohol on HDL-C as found by the MR studies in our review is in line with short-term RCTs, whereas these RCTs did not find an effect on other cardiometabolic risk factors such as blood pressure [8, 9, 11]. However, genetically predicted alcohol consumption as used in MR is thought to reflect lifetime alcohol consumption [20], while the majority of RCTs investigated effects of changing alcohol consumption over a timespan of weeks or months only.

We observed substantial differences in methodological quality between the included studies. A full IV analysis—which is needed to estimate the size of the causal effect—was performed in 71% of the studies. In some studies, the choice not to perform a full IV analysis was made deliberately, as the commonly used methods to perform an IV analysis in MR assume linearity, whereas the associations with cardiometabolic health in observational studies are often J- or U-shaped.

Half of the studies verified all three key assumptions. All studies verified whether the genetic instrument was a suitable instrument for the exposure (first assumption). Typically, the variance in alcohol consumption that is explained by the genetic variants is small, with the exception of a functional variant in *ALDH2* which explains quite a high proportion of the variance in alcohol consumption in Asian populations and—to a lesser extent—a functional variant in *ADH1B* for European populations. Hence, large sample sizes are required to perform sufficiently powered MR analyses, especially in European populations. Evidence for a harmful effect of alcohol consumption is more apparent in more recent studies, which tend to be larger and hence better-powered.

The MR design could be quite suitable for an exposure like alcohol consumption, which is associated with many other factors (such as social economic status and diet) and disentangling these associations can be very challenging in conventional analyses. As explained earlier in this paper, the use of a negative control is a comprehensible method to verify whether the genetic proxy exerts its effect on the outcome exclusively through the exposure (third assumption) and thus whether the association between exposure and outcome is truly causal. Studies based on populations of Asian ancestry usually performed stratified analyses by sex, as in some East Asian regions women tend to abstain from alcohol due to cultural reasons [39] and this provides a convenient natural control group. In Europe, this cultural phenomenon does not apply, and therefore in these studies similar associations in both men and women were assumed. Here, validation of the third assumption was most often literature-based [42, 44–46] or non-drinkers were used as negative controls [43]. Additionally, positive controls (i.e., testing the association between SNPs and an outcome for which clear associations with alcohol consumption already exist) could possibly strengthen validation of the key assumptions.

### Strengths and limitations

With the number of MR studies expanding rapidly, we aimed to provide a status update on the research conducted so far in the field of alcohol and cardiometabolic disease. Although no formal data extraction or quality assessment tool for MR studies is available yet, we used a comprehensive data extracting protocol that has been proposed as guidance by experts in the field [24]. As for quality assessment, we tried to capture the essential elements of the MR design, to be able to make comparisons between studies regarding these criteria.

A limitation of our work is that we were not able to meta-analyze the results due to the large methodological heterogeneity in analyzing techniques, genetic instrumental variables and units between studies, but were limited to a qualitative description of results. Moreover, we might not have captured all MR studies performed in the field, because our search strategy was limited to studies claiming to have used the MR design or instrumental variable analysis in title, abstract or keywords. Lastly, conclusions may only be generalizable to populations of European and Asian descent, since individuals of other ancestries were not investigated in the included studies.

### Recent advancements in the field of MR

MR is a relatively new epidemiological study design that recently gained popularity. As such, it is a dynamic study field in which new insights in methodology and more advanced statistical techniques are emerging regularly. As GWAS are relatively common now and have been published on many different phenotypes in extremely large sample sizes, it is now possible to identify a multitude of SNPs associated with alcohol consumption at genome-wide significance, without the need to know the biological mechanism behind this association [55]. The use of multiple SNPs allows for the application of a wide range of sensitivity analyses that have been developed to assess the robustness of findings to pleiotropy and invalidity of the genetic instrument [27]. Furthermore, if the simultaneous use of multiple SNPs increases the phenotypic variance explained, it contributes to more powerful MR analyses. The possibility of combining multiple datasets in the two-sample MR design further increases power. The most recent MR studies in this review are indeed all two-sample MR studies, using GWAS to select their SNPs and applying the new methodology to ensure valid results.

Another methodological advancement that is important for alcohol research has been made on the assessment of non-linearity in MR. Traditional statistical MR methods such as 2SLS assume a linear relation between exposure and outcome. Recently, the LACEs method has been developed to examine non-linearity [28, 46]. Especially if the observational literature points towards a non-linear association as is the case for alcohol and cardiometabolic outcomes, it is of great additional value to assess potential non-linearity with the MR method. In this review, two studies have adopted the LACEs method to address potential non-linearity, of which one found evidence for a non-linear trend where the other did not [35, 46]. The other three studies that used a different method also reported mixed results [41, 43, 47]. Further development of methods to study non-linearity will probably lead to a more frequent use of these analyzing techniques in future work.

It was difficult to determine whether it is valid to draw conclusions from many of the included MR studies presented because of the poor (reporting of the) assessment of the MR assumptions. This poor reporting in MR studies has previously been observed by others as well and has led to the development of the STROBE-MR guidelines [56]. We highly recommend future MR studies to use this guideline for their reporting, such that readers can cautiously interpret the results taking potential bias from violation of the assumptions into account. In addition, the recently published guideline on the methodology of MR studies that will be updated regularly can be used by researchers to select the best and most up-to-date methodology for their MR study [27].

### Triangulation of evidence

Since the emergence of the MR design, the scientific world has been eager to adopt this research technique as a new strategy to address causality. However, MR studies have their own potential sources of bias including bias from invalid instruments as we indicated before, but also for example selection bias due to study sampling and bias from residual population stratification [27, 57]. There has been growing consensus that evidence from MR studies should be regarded in the context of available evidence from other epidemiological study designs, such as (prospective) observational studies and RCTs before conclusions on causality can be drawn [54, 58, 59]. This triangulation of evidence approach relies on evaluating findings from different study types that have different and unrelated sources of bias [58]. If findings are in line across different study types, causal inference is strengthened [58, 60]. For alcohol research, the majority of evidence is available from observational studies and, to a lesser extent, from short-term RCTs. In addition to future long-term intervention studies, we think that MR studies add a new dimension to the body of evidence as well.

## Conclusions

The current MR studies on alcohol consumption and cardiometabolic health show substantial heterogeneity in the chosen methodology and in the reporting of the methodological quality. This makes it difficult to draw firm conclusions on the causal role of alcohol-in-moderation on cardiometabolic health. Part of this heterogeneity can probably be explained by MR being a relatively new and dynamic field in which new methodological insights are provided on a regular basis. We expect that with the continuous advancements in the field of MR, the role of MR in triangulation of evidence becomes more important, although it should not yet be considered a replacement for a long-term RCT. The last word has not been said yet on the alcohol-in-moderation debate and we expect that future MR studies, adopting the most recent advancements regarding instrument selection and non-linearity methodology, will further substantiate this discussion.

## Supplementary Information

Below is the link to the electronic supplementary material.

Supplementary file1 (DOCX 338 kb)

## Data Availability

All data are available from the corresponding author upon reasonable request.
