# Supplementary material for: Alcohol consumption in relation to cardiovascular diseases and mortality: a systematic review of Mendelian randomization studies
Source: Eur J Epidemiol. 2021 Aug 22;37(7):655–69. doi: 10.1007/s10654-021-00799-5 (PMC9329419; doi:10.1007/s10654-021-00799-5)
Supplement: Supplementary file 1 — Supplementary file1 (DOCX 338 kb) [file 10654_2021_799_MOESM1_ESM.docx]

**Supplementary materials**

**Alcohol consumption in relation to cardiovascular diseases and mortality: a systematic review of Mendelian randomization studies**

European Journal of Epidemiology

Inge A.T. van de Luitgaarden, Sabine van Oort, Emma J. Bouman, Linda J. Schoonmade, Ilse C. Schrieks, Diederick E. Grobbee, Yvonne T. van der Schouw, Susanna C. Larsson, Stephen Burgess, Adriana J. van Ballegooijen, N. Charlotte Onland-Moret, Joline W.J. Beulens

***Correspondence****:*

*Inge van de Luitgaarden; Julius Center for Health Sciences and Primary Care, University Medical Center Utrecht, Utrecht University, Utrecht, the Netherlands*

*E-mail:* [*I.A.T.vandeLuitgaarden@umcutrecht.nl*](mailto:I.A.T.vandeLuitgaarden@umcutrecht.nl)

| **List of Supplementary Materials** | **Page** |
| --- | --- |
| Supplementary Methods 1. Search string | 2 |
| Supplementary Table 1. Methodological quality assessment of the 24 included Mendelian randomization studies. | 3 |
| Supplementary Table 2. Overview of the associations of genetically higher alcohol consumption with anthropometric measures in the included Mendelian randomization studies | 7 |
| Supplementary Table 3. Overview of the associations of genetically higher alcohol consumption with blood pressure in the included Mendelian randomization studies | 9 |
| Supplementary Table 4. Overview of the associations of genetically higher alcohol consumption with lipids in the included Mendelian randomization studies | 11 |
| Supplementary Table 5. Overview of the associations of genetically higher alcohol consumption with diabetes-related measures in the included Mendelian randomization studies | 14 |
| Supplementary References | 16 |

**Supplementary Methods 1. Search string**

| **Database** | **Result** |
| --- | --- |
| PubMed | 301 |
| Embase | 413 |
| Scopus | 454 |
| Total | 1168 |
| **Total without duplicates** | **623** |

**History PubMed November 16, 2020**

| **Search** | **PubMed Query – November 16, 2020** | **Items found** |
| --- | --- | --- |
| #3 | #1 AND #2 | 301 |
| #2 | "Mendelian Randomization Analysis"[Mesh] OR mendelian[tiab] | 13,437 |
| #1 | "Alcohols"[Mesh] OR "Alcohol Drinking"[Mesh] OR "Alcoholic Beverages"[Mesh] OR "Alcoholism"[Mesh] OR alcohol*[tiab] OR drinking[tiab] | 1,025,290 |

**History Embase.com November 16, 2020**

| **Search** | **Embase.com Query - November 16, 2020** | **Items found** |
| --- | --- | --- |
| #3 | #1 AND #2 | 413 |
| #2 | 'instrumental variable analysis'/exp OR mendelian:ab,ti,kw | 17,891 |
| #1 | 'alcohol'/exp OR 'alcohol abuse'/exp OR 'alcoholic beverage'/exp OR 'drinking behavior'/exp OR 'drinking'/exp OR alcohol*:ab,ti,kw OR drinking:ab,ti,kw | 735,105 |

**History Scopus November 16, 2020**

| **Search** | **Scopus Query - November 16, 2020** | **Items found** |
| --- | --- | --- |
| #3 | #1 AND #2 | 454 |
| #2 | TITLE-ABS-KEY (mendelian) | 18,182 |
| #1 | TITLE-ABS-KEY (alcohol* OR drinking) | 1,070,533 |

**Google Scholar search string**

mendelian alcohol*|drinking

**Supplementary Table 1. Methodological quality assessment of the 24 included Mendelian randomization studies**

| *Study* | *Prioritizing IV* | *IV analysis* | *MR assumption 1* | *MR assumption 2* | *MR assumption 3* | *Sensitivity analyses* | *Non-linearity* |
| --- | --- | --- | --- | --- | --- | --- | --- |
| Chen (2008) (1) | Literature-based, functional SNP | 2SLS IV analysis | F-statistic tested: ranging from 42 to 1962 | Tested, no associations IV with potential confounders, except from BMI in men in the two largest studies | Negative controls (women), showing that alcohol intake nor blood pressure varied noticeably by genotype in women | None performed | Not tested |
| Au Yeung (2013) (2) | Literature-based, functional SNP | Linear regression for allele carriage; no full IV analysis | F-statistic tested: 75.0 (r^2^= 3.0%) | Tested, no associations IV – potential confounders | Negative controls (never or occasional drinkers)  Partial, but no full attenuation of IV – outcome association after controlling for alcohol consumption. | Analysis with never/occasional drinkers only (negative controls) | Not tested |
| Au Yeung (2013) (3) | Literature-based, functional SNP | 2SLS IV analysis (CVD risk factors) or IV probit regression (CVD morbidity) | F-statistic tested: 74.6 (r^2^= 3.0%) | Tested, no associations IV – potential confounders | Analysis with negative controls is reported, but data is not shown | Exclusion of   1. former drinker 2. heavy drinkers | Not tested |
| Lawlor (2013) (4) | Literature-based, functional SNPs | Control function method | F-statistic tested: 29 | Tested, no association IV – potential confounders. | Assumed, not tested | 1. Genetic variants as weighted allele score 2. 2SLS and GMM analysis 3. Interaction with age and sex tested 4. Analysis in elderly only 5. Association of genetic variants with beverage type 6. Gene-alcohol interaction | Not tested |
| Holmes (2014) (5) | Literature-based, functional SNP | Meta-regression for allele carriage; no full IV analysis | Tested, no F-statistic provided | Tested, no association IV – potential confounders | Negative controls (non-drinkers) | Analysis was repeated in strata of alcohol intake (none; light-to-moderate, heavy) | Analysis was repeated in strata of alcohol intake (none; light-to-moderate, heavy) |
| Silverwood (2014) (6) | Literature-based, functional SNP | 2SLS and LACE method | Tested, no F-statistic provided | Assumed, as this was a continuation of a previous analysis in the Alcohol-ADH1B Consortium (Holmes (2014)) | Assumed, as this was a continuation of a previous analysis in the Alcohol-ADH1B Consortium (Holmes (2014)) | Analysis to determine the ‘optimal’ amount of alcohol consumption regarding the investigated outcomes. | The LACE method was used to examine the existence of non-linear causality. |
| Cho (2015) (7) | Literature-based, functional SNP | 2SLS IV analysis | F-statistic tested:  262 (men)  38 (women) | Tested, no associations IV – potential confounders.  IV analysis adjusted for potential confounders | Negative controls (male never drinkers, and women) | Additional adjustment for smoking in analysis male non-drinkers.  Population-level causal effects were assessed using the interaction between genetic variant and sex as instrument. | Not tested |
| Taylor (2015) (8) | Literature-based, functional SNP | Linear regression for allele carriage; no full IV analysis | Tested, no F-statistic provided | Not tested | Negative controls (women) | Analyses stratified for diabetes status | Not tested |
| Jee (2016) (9) | Literature-based, functional SNP | Linear regression for allele carriage and triangulation approach.; no full IV analysis | F-statistic tested:  302.6 (men)  44.3 (women) | Tested, no association IV – potential confounders.  Analysis adjusted for potential confounders. | Negative controls (women) | Exclusion of   1. Heavy drinkers 2. Elderly | Not tested |
| Tabara (2016) (10) | Literature-based, functional SNP | Linear regression for allele carriage; no full IV analysis | Tested, no F-statistic provided | Tested, no association IV – potential confounders, except from glucose in men.  Analysis adjusted for potential confounders | Not reported | None performed | Not tested |
| Vu (2016) (11) | Unweighted genetic risk score, based on GWASs | 2SLS IV analysis | Tested, F-statistic range: 10.9-13.5 | Tested, no association IV – potential confounders | Tested for linkage disequilibrium (r^2^ > 0.2) between IV and outcome-related loci | Exclusion of:   1. Heavy drinkers 2. Never drinkers 3. Never and heavy drinkers 4. Never and former drinkers | Non-linear relationships were evaluated in the second stage of the 2SLS using the predicted alcohol consumption categories in quartiles.  Sensitivity analyses were performed excluding heavy drinkers to examine the effect of alcohol within the low-to-moderate range. |
| Almeida (2017) (12) | Literature-based, functional SNP | Cox regression for allele carriage; no full IV analysis | Tested, no F-statistic provided | Tested, no associations IV – potential confounders | Assumed, not tested | Cox regression adjusted for interaction between alcohol consumption group membership and being a carrier | Not tested |
| Tabara (2017) (13) | Literature-based, functional SNP | Linear regression for allele carriage; no full IV analysis | Tested, no F-statistic provided | Tested, no association IV – potential confounders, except from blood pressure.  Analysis adjusted for potential confounders | Not reported | None performed | Not tested |
| Cho (2018) (14) | Literature-based, functional SNPs | 2SLS IV analysis | F-statistic tested: 47.9 | Tested, no association IV – potential confounders except for BMI in women.  IV analysis adjusted for potential confounders. | Negative controls (women) | 1. Analysis with ALDH2 + ADH1B as unweighted genetic risk score | Not tested |
| Christensen (2018) (15) | Literature-based, functional SNPs | 2SLS IV analysis | F-statistic tested: 22.0 | Tested, no association IV – potential confounders.  IV analysis adjusted for age and sex. | Assumed, not tested | None performed | Not tested |
| Millwood (2019) (16) | Literature-based, functional SNPs | Inverse-variance weighted meta-analysis of the within-area slopes of genotype predicted alcohol consumption categories (x-axis) plotted against means or log RRs in each consumption category (y-axis) | Tested, no F-statistic provided | Tested, no association IV – potential confounders except for age and education in men and smoking status in women.  All analyses adjusted for age. Sensitivity analysis adjusted for additional confounders in men. | Negative controls (women) | 1) Inclusion of former drinkers  2) Additional adjustment for education, income and smoking in men  3) Analyses also conducted for individual SNPs to obtain per-allele effects | Results presented in genotype predicted alcohol consumption categories to evaluate potential non-linear effects.  Comparison of observed genetic epidemiological results with the expected results if moderate alcohol intake would be 1) protective or 2) not protective. |
| Peng (2019) (17) | Literature-based, functional SNP | 2SLS (diabetes-related traits) and GMM (diabetes) IV analysis  LACE method | F-statistic tested:  169.5 (men)  80.6 (women) | Tested, no association IV – potential confounders.  Main analysis adjusted for potential confounders. | Negative controls (non-drinkers and women) | 1. Not excluding participants drinking other types of alcoholic beverages than liquor 2. Complete case analysis | The LACE method was used to examine the existence of non-linear causality. |
| Zhao (2019) (18) | Literature-based, functional SNP | 2SLS IV analysis | Tested, no F-statistic provided | Tested, no association IV – potential confounders | Negative controls (women) | None performed | Not tested |
| Larsson (2020) (19) | Combination of genome-wide significant SNPs based on GWAS  Secondary analysis:  Literature-based, functional SNP | IVW method and multivariable MR analyses | Selection of strongly and robustly associated SNPs from GWAS  F-statistic reported: 29 | Tested, association IV (risk score) – smoking initiation found, analysis adjusted for this confounder in multivariable MR analyses.  Secondary analysis:  Association IV (single SNP) – education. No adjustment. | Tested with formal analyses: Weighted median method, MR-Egger and MR-PRESSO. | 1. Analyses to explore potential pleiotropy (see analyses under assumption 3) 2. Multivariable MR analyses 3. Analyses in never-smokers only 4. Analyses in European-only datasets 5. Analyses with exclusion of ADH1B (rs1229984) from risk score | Not tested |
| Jiang (2020) (20) | Combination of genome-wide significant SNPs based on GWAS | IVW method and multivariable MR analyses | Selection of strongly and robustly associated SNPs from GWAS  r^2^=0.16% | Analysis adjusted for smoking (in multivariable MR analyses) | Tested with formal analyses: MR-Egger, simple median, weighted median, penalized weighted median, maximum likelihood methods and MR-PRESSO | 1. Analyses to explore potential pleiotropy (see analyses under assumption 3) 2. Exclusion of SNPs associated with smoking phenotypes 3. Leave-one-out analysis | Not tested |
| Van Oort (2020) (21) | Combination of genome-wide significant SNPs based on GWAS | IVW method and multivariable MR analyses | Selection of strongly and robustly associated SNPs from GWAS  r^2^=0.2% | Analysis adjusted for smoking initiation (in multivariable MR analyses) | Tested with formal analyses: Weighted median method, MR-Egger and MR-PRESSO. | 1. Analyses to explore potential pleiotropy (see analyses under assumption 3) 2. Exclusion of pleiotropic SNPs 3. Multivariable MR analyses | Not tested |
| Yuan (2020) (22) | Combination of genome-wide significant SNPs based on GWAS | IVW method and multivariable MR analyses | Selection of strongly and robustly associated SNPs from GWAS | Analysis adjusted for BMI (in multivariable MR analyses) | Tested with formal analyses: Weighted median method and MR-Egger | 1. Analyses to explore potential pleiotropy (see analyses under assumption 3) 2. Multivariable MR analyses 3. Replication of analyses in other consortium | Not tested |
| Van Oort (2020) (23) | Combination of genome-wide significant SNPs based on GWAS | IVW method | Selection of strongly and robustly associated SNPs from GWAS  r^2^=0.2% | In case of significant associations, adjustment for diabetes and CVD (Multivariable MR) | Tested with formal analyses: Weighted median method, contamination mixture method, MR-Egger and MR-PRESSO | 1. Analyses to explore potential pleiotropy (see analyses under assumption 3) | Not tested |
| Van Oort (2020) (24) | Combination of genome-wide significant SNPs based on GWAS | IVW method | Selection of strongly and robustly associated SNPs from GWAS  r^2^=0.2% | Multivariable MR for other exposures, not relevant for alcohol consumption | Tested with formal analyses: Weighted median method, MR-Egger and MR-PRESSO | 1. Analyses to explore potential pleiotropy (see analyses under assumption 3) 2. Analyses with self-reported hypertension as outcome | Not tested |

Abbreviations: CVD = cardiovascular disease; GMM = general methods of moments; GWAS = genome-wide association study; IV = instrumental variable; IVW = inverse-variance weighted; LACE = localized average causal effect; MR = Mendelian randomization; MR-PRESSO = Mendelian Randomization Pleiotropy RESidual Sum and Outlier ; SNP = single nucleotide polymorphism; 2SLS = 2 stage least squares; RR = risk ratio. First assumption: the genetic variant is associated with alcohol consumption. Second assumption: the genetic variant is not associated with any confounder of the alcohol consumption-outcome association. Third assumption: the genetic variant does not affect the outcome, except possibly via its association with alcohol consumption.

**Supplementary table 2. Overview of the associations of higher genetically predicted alcohol consumption with anthropometric measures in the included Mendelian randomization studies**

| **Outcome and study** | **Ancestry** | **IV** | **Effect measure and unit** | **Association with outcome** | | |
| --- | --- | --- | --- | --- | --- | --- |
|  |  |  |  | **Total** | **Men** | **Women** |
| **Body mass index** | | | | | | |
| AuYeung (2013) (2) | Asian | No | kg/m^2^ for GA vs. AA genotype |  | -0.003 (-0.34; 0.33) |  |
| Cho (2015) (7) | Asian | Yes | kg/m^2^ per g/day | 0.004 (-0.017; 0.024) | 0.012 (-0.001; 0.025) | 0.098 (-0.079; 0.274) |
| Holmes (2014) * (5) | European | No | kg/m^2^  GG vs. AA or AG | **0.17 (0.10; 0.24)** |  |  |
| Lawlor (2013) (4) | European | Yes | kg/m^2^ per doubling of alcohol consumption in current drinkers | **1.37 (0.59; 2.15)** |  |  |
| Millwood (2019) (16) | Asian | Yes | kg/m^2^ per 280 g/week |  | **0.37 (0.28; 0.46)** | **0.11 (0.03; 0.19)** |
| Peng (2019) (17) | Asian | Yes | kg/m^2^ for 22 g increase in log-transformed alcohol |  | **0.57 (0.28; 0.87)** | -0.04 (-1.02; 0.94) |
| Silverwood (2014) * (6) | European | Yes | kg/m^2^ for 1 unit increase in log-transformed alcohol | **0.70 (0.2; 1.2)** |  |  |
| Taylor (2015) (8) | Asian | No | kg/m^2^ per copy of G allele |  | 0.21 (-0.07; 0.49) | 0.05 (-0.19; 0.28) |
| Zhao (2019) (18) | Asian | Yes | kg/m^2^ for alcohol vs. no alcohol | 0.61 (-0.37; 1.58) | 1.43 (-0.24; 1.53) | 0.25 (-2.64; 3.41) |
|  |  |  |  |  |  |  |
| **Weight** |  |  |  |  |  |  |
| Millwood (2019) (16) | Asian | Yes | kg per 280 g/week |  | **1.39 (1.11; 1.67)** | **0.57 (0.36; 0.78)** |
| Zhao (2019) (18) | Asian | Yes | kg for alcohol vs. no alcohol | **3.17 (0.38; 5.96)** | **2.89 (0.19; 5.58)** | 3.92 (-4.22; 12.07) |
|  |  |  |  |  |  |  |
| **Waist circumference** | |  |  |  |  |  |
| Cho (2015) (7) | Asian | Yes | cm per g/day | 0.02 (-0.03; 0.08) | **0.06 (0.03; 0.09)** | 0.43 (-0.06; 0.91) |
| Holmes (2014) * (5) | European | No | cm for GG vs. AA or AG | **0.34 (0.10; 0.58)** |  |  |
| Millwood (2019) (16) | Asian | Yes | cm per 280 g/week |  | **1.87 (1.60; 2.14)** | **0.43 (0.21; 0.65)** |
| Peng (2019) (17) | Asian | Yes | cm for 22 g increase in log-transformed alcohol |  | **2.37 (1.47; 3.24)** | -0.63 (-3.31; 2.01) |
| Silverwood (2014) * (6) | European | Yes | cm for 1 unit increase in log-transformed alcohol | **2.80 (1.3; 4.4)** |  |  |
| Zhao (2019) (18) | Asian | Yes | cm for alcohol vs. no alcohol | 2.58 (0.00; 5.16) | 1.41 (-1.07; 3.89) | 3.74 (-4.40; 11.88) |
|  |  |  |  |  |  |  |
| **Waist-to-hip ratio** |  |  |  |  |  |  |
| Cho (2015) (7) | Asian | Yes | beta coefficient per g/day | **0.0005 (0.0001; 0.0009)** | **0.0006 (0.0004; 0.0008)** | 0.0012 (-0.0023; 0.0047) |
| Millwood (2019) (16) | Asian | Yes | beta coefficient per 280 g/week |  | **1.51 (1.33; 1.70)** | 0.13 (-0.03; 0.29) |
| Peng (2019) (17) | Asian | Yes | beta coefficient for 22 g increase in log-transformed alcohol |  | 0.016 (0.00; 0.023) | 0.003 (-0.016; 0.023) |

* These two studies had overlapping study populations

The results presented here are the results of the linear analyses. Detrimental associations have been displayed in **bold red**.

Abbreviations: IV = instrumental variable analysis

**Supplementary Table 3. Overview of the associations of genetically higher alcohol consumption with blood pressure in the included Mendelian randomization studies**

| **Outcome and study** | **Ancestry** | **IV** | **Effect measure and unit** | **Association with outcome** | | |
| --- | --- | --- | --- | --- | --- | --- |
|  |  |  |  | **Total** | **Men** | **Women** |
| **Hypertension** | | | | | | |
| Chen (2008) (1) | Asian | No | OR for *1*1 homozygotes vs. *2*2 homozygotes |  | **2.42 (1.66; 3.55)** | No association |
| Cho (2015) (7) | Asian | Yes | OR per g/day | **1.03 (1.00; 1.06)** | **1.02 (1.01;1.03)** | 1.04 (0.92;1.18) |
| Cho (2018) * (14) | Asian | Yes | OR per g/day | **1.04 (1.01; 1.06)** | 1.02 (1.00; 1.04) | 1.24 (0.97; 1.57) |
| Cho (2018) * † (14) | Asian | Yes | OR per g/day | 1.02 (1.00; 1.05) | **1.02 (1.00; 1.04)** | 0.98 (0.82; 1.18) |
| Holmes (2014) * (5) | European | No | OR GG vs. AA or AG | **1.12 (1.01; 1.23)** |  |  |
| Van Oort (2020) (24) | European | Yes | OR per 1-SD increase in log-transformed alcoholic drinks/week | **1.28 (1.07; 1.52)** |  |  |
| Zhao (2019) (18) | Asian | Yes | OR alcohol vs. no alcohol | **1.20 (1.04; 1.39)** | **1.19 (1.04; 1.36)** | No association |
|  |  |  |  |  |  |  |
| **Systolic blood pressure** |  |  |  |  |  |  |
| AuYeung (2013) ‡ (3) | Asian | Yes | mmHg per 10 g/day |  | 1.00 (-0.74; 2.74) |  |
| AuYeung (2013) ‡ (2) | Asian | No | mmHg for GA vs. AA genotype |  | 1.34 (-0.90; 3.59) |  |
| Chen (2008) (1) | Asian | Yes | mmHg per g alcohol/day |  | **0.24 (0.16;0.32)** | No association |
| Cho (2015) * (7) | Asian | Yes | mmHg per g/day | **0.20 (0.09; 0.32)** | **0.16 (0.09; 0.23)** | -0.35 (-1.29; 0.59) |
| Cho (2018) * (14) | Asian | Yes | mmHg per g/day | **0.23 (0.07; 0.40)** | 0.12 (-0.02; 0.26) | 1.71 (-0.13; 3.56) |
| Cho (2018) *† (14) | Asian | Yes | mmHg per g/day | 0.12 (-0.03; 0.27) | 0.07 (-0.06; 0.20) | 0.63 (-0.56; 1.82) |
| Holmes (2014) \|\| (5) | European | No | mmHg. GG vs. AA or AG | **0.88 (0.56; 1.19)** |  |  |
| Larsson (2020) (19) | European | Yes | mmHg per 1-SD increase in log-transformed drinks/week | **0.12 (0.05; 0.20)** |  |  |
| Lawlor (2013) (4) | European | Yes | mmHg per doubling of alcohol consumption in current drinkers | 0.94 (-3.03; 4.91) |  |  |
| Millwood (2019) (16) | Asian | Yes | mmHg per 280 g/week |  | **4.3 (3.7; 4.9)** | **-0.6 (-1.0; -0.1)** |
| Peng (2019) (17) | Asian | Yes | mmHg for 22 g increase in log-transformed alcohol |  | **2.91 (1.06; 4.76)** | -1.97 (-8.12; 4.18) |
| Silverwood (2014) \|\| (6) | European | Yes | mmHg for 1 unit increase in log-transformed alcohol | **5.20 (3.20; 7.30)** |  |  |
| Taylor (2015) (8) | Asian | No | mmHg per copy of G allele |  | **2.26 (3.72; 0.79)** | -0.53 (-1.82; 0.75) |
| Zhao (2019) (18) | Asian | Yes | mmHg for alcohol vs. no alcohol | **9.46 (3.84; 15.08)** | **8.28 (3.22; 13.35)** | 13.44 (-4.18; 31.07) |
|  |  |  |  |  |  |  |
| **Diastolic blood pressure** | |  |  |  |  |  |
| AuYeung (2013) ‡ (3) | Asian | Yes | mmHg per 10 g/day |  | **1.15 (0.23; 2.07)** |  |
| AuYeung (2013) ‡ (2) | Asian | No | mmHg for GA vs. AA genotype |  | 0.47 (-0.71; 1.64) |  |
| Chen (2008) (1) | Asian | Yes | mmHg per g alcohol/day |  | **0.16 (0.11;0.21)** | No association |
| Cho (2015) * (7) | Asian | Yes | mmHg per g/day | **0.10 (0.03; 0.18)** | **0.09 (0.04; 0.14)** | -0.11 (-0.70; 0.48) |
| Cho (2018) * (14) | Asian | Yes | mmHg per g/day | **0.12 (0.02; 0.23)** | 0.06 (-0.03; 0.15) | 0.89 (-0.14; 1.91) |
| Cho (2018) *† (14) | Asian | Yes | mmHg per g/day | **0.11 (0.01; 0.20)** | 0.06 (-0.02; 0.15) | 0.53 (-0.19; 1.25) |
| Holmes (2014) (5) | European | No | mmHg for GG vs. AA or AG | 0.08 (-0.10; 0.25) |  |  |
| Larsson (2020) (19) | European | Yes | mmHg per 1-SD increase in log-transformed drinks/week | **0.15 (0.05; 0.25)** |  |  |
| Lawlor (2013) (4) | European | Yes | mmHg per doubling of alcohol consumption in current drinkers | 0.23 (-1.95; 2.41) |  |  |
| Millwood (2019) (16) | Asian | Yes | mmHg per 280 g/week |  | **3.07 (2.75; 3.40)** | 0.11 (-0.14; 0.36) |
| Peng (2019) (17) | Asian | Yes | mmHg for 22 g increase in log-transformed alcohol |  | **3.03 (1.87; 4.19)** | 1.54 (-2.04; 5.11) |
| Taylor (2015) (8) | Asian | No | mmHg per copy of G allele |  | **1.52 (0.58; 2.45)** | -0.19 (-0.91; 0.53) |
| Zhao (2019) (18) | Asian | Yes | mmHg for alcohol vs. no alcohol | **7.50 (3.73; 11.26)** | **6.72 (3.28; 10.15)** | 10.28 (-1.41; 21.96) |

* These two studies had overlapping study populations

† Unweighted genetic risk score of 2 SNPs as instrument instead of 1 SNP as in line above

‡ Same study population

|| Overlapping study population

The results presented here are the results of the linear analyses. Detrimental associations have been displayed in **bold red** and beneficial associations in **bold blue**.

Abbreviations: IV= instrumental variable analysis

**Supplementary Table 4. Overview of the associations of genetically higher alcohol consumption with lipids in the included Mendelian randomization studies**

| **Outcome and study** | **Ancestry** | **IV** | **Effect measure and unit** | **Association with outcome** | | |
| --- | --- | --- | --- | --- | --- | --- |
|  |  |  |  | **Total** | **Men** | **Women** |
|  |  |  |  |  |  |  |
| **Total cholesterol** |  |  |  |  |  |  |
| Cho (2015) (7) | Asian | Yes | mg/dL per g/day alcohol | 0.04 (-0.20; 0.27) | -0.04 (-0.20; 0.12) | -0.897 (-2.82; 1.02) |
| Peng (2019) (17) | Asian | Yes | mmol/L for 22 g increase in log-transformed alcohol |  | **0.15 (0.06; 0.25)** | -0.04 (-0.33; 0.26) |
| Taylor (2015) (8) | Asian | No | mmol/L per copy of G allele |  | **0.12 (0.02; 0.21)** | 0.01 (-0.06; 0.08) |
| Vu (2016) (11) | European | Yes | mg/dL per quartile predicted alcohol consumption (Q3 vs. Q1) | **-7.71 (-13.26; -2.15)** |  |  |
| Zhao (2019) (18) | Asian | Yes | beta coefficient (unit unknown) alcohol vs. no alcohol | 0.20 (-0.08; 0.48) | 0.15 (-0.10; 0.40) | 0.31 (-0.57; 1.18) |
|  |  |  |  |  |  |  |
| **HDL cholesterol** |  |  |  |  |  |  |
| AuYeung (2013) * (2) | Asian | No | mmol/L, GA vs. AA genotype |  | **0.05 (0.01; 0.09)** |  |
| AuYeung (2013) * (3) | Asian | Yes | mmol/L per 10 g/day |  | **0.05 (0.02; 0.08)** |  |
| Cho (2015) (7) | Asian | Yes | mg/dL per g/day alcohol | **0.17 (0.10; 0.23)** | **0.17 (0.12; 0.22)** | 0.27 (-0.28; 0.81) |
| Holmes (2014) † (5) | European | No | mmol/L. GG vs. AA or AG | 0.004 (-0.003; 0.012) |  |  |
| Larsson (2020) (19) | European | Yes | mmol/L per 1-SD increase in log-transformed drinks/week | **0.39 (0.25; 0.53)** |  |  |
| Lawlor (2013) (4) | European | Yes | % HDL per doubling of alcohol consumption in current drinkers | 1.5 (-4.5; 7.4) |  |  |
| Millwood (2019) (16) | Asian | Yes | mmol/L per 280 g/week |  | **0.16 (0.13; 0.19)** | 0.00 (-0.03; 0.03) |
| Peng (2019) (17) | Asian | Yes | mmol/L for 22 g increase in log-transformed alcohol |  | 0.04 (-0.002; 0.08) | 0.04 (-0.07; 0.15) |
| Silverwood (2014) † (6) | European | Yes | mmol/L for 1 unit increase in log-transformed alcohol | -0.02 (-0.07; 0.03) |  |  |
| Tabara (2016) (10) | Asian | No | mg/dL per *1 allele vs. *2*2 homozygotes |  | **2.87 (1.83; 3.91)** | 0.74 (-0.48; 1.96) |
| Tabara (2017) (13) | Asian | No | mg/dL per *1 allele vs. *2*2 homozygotes |  | **2.89 (2.09; 3.69)** | **1.00 (0.41; 1.59)** |
| Taylor (2015) (8) | Asian | No | mmol/L per copy of G allele |  | **0.05 (0.01; 0.09)** | 0.03(-0.01; 0.06) |
| Vu (2016) † (11) | European | Yes | ln mg/dL per quartile predicted alcohol consumption (Q3 vs. Q1) | 0.04 (0.00; 0.07) |  |  |
| Zhao (2019) (18) | Asian | Yes | beta coefficient (unit unknown) alcohol vs. no alcohol | -0.47 (-1.04; 0.09) | -0.61 (-1.37; 0.14) | 0.01 (-0.25; 0.28) |
|  |  |  |  |  |  |  |
| **LDL cholesterol** |  |  |  |  |  |  |
| AuYeung (2013) * (2) | Asian | No | mmol/L, GA vs. AA genotype |  | 0.05 (-0.02; 0.12) |  |
| AuYeung (2013) * (3) | Asian | Yes | mmol/L per 10 g/day |  | 0.03 (-0.03; 0.08) |  |
| Cho (2015) (7) | Asian | Yes | mg/dL per g/day alcohol | **-0.36 (-0.58; -0.15)** | **-0.41 (-0.55; -0.26)** | -1.01 (-2.70; 0.69) |
| Larsson (2020) (19) | European | Yes | mmol/L per 1-SD increase in log-transformed drinks/week | -0.01 (-0.18; 0.16) |  |  |
| Millwood (2019) (16) | Asian | Yes | mmol/L per 280 g/week |  | -0.03 (-0.1; 0.03) | -0.06 (-0.12; 0.01) |
| Tabara (2016) (10) | Asian | No | mg/dL per *1 allele vs. *2*2 homozygotes |  | **-7.85 (-9.93; -5.77)** | -0.61 (-2.98; 1.76) |
| Tabara (2017) (13) | Asian | No | mg/dL per *1 allele vs. *2*2 homozygotes |  | **-2.65 (-4.59; -0.71)** | -1.19 (-2.41; 0.03) |
| Taylor (2015) (8) | Asian | No | mmol/L per copy of G allele |  | -0.01 (-0.09; 0.07) | -0.03 (-0.09; 0.04) |
| Vu (2016) (11) | European | Yes | mg/dL per quartile predicted alcohol consumption (Q3 vs. Q1) | **-6.87 (-12.24; -1.50)** |  |  |
| Zhao (2019) (18) | Asian | Yes | beta coefficient (unit unknown) alcohol vs. no alcohol | -0.03 (-0.28; 0.22) | -0.18 (-0.40; 0.04) | 0.41 (-0.38; 1.22) |
|  |  |  |  |  |  |  |
| **Triglycerides** |  |  |  |  |  |  |
| AuYeung (2013) * (2) | Asian | No | mmol/L for GA vs. AA genotype |  | 0.002 (-0.14; 0.14) |  |
| AuYeung (2013) * (3) | Asian | Yes | log mmol/L per 10 g/day |  | 0.03 (-0.01; 0.08) |  |
| Cho (2015) (7) | Asian | Yes | log mg/dL per g/day alcohol | **0.003 (0.002; 0.005)** | **0.002 (0.001; 0.003)** | -0.007 (-0.018; 0.004) |
| Holmes (2014) † (5) | European | No | % triglycerides for GG vs. AA or AG | **-1.61 (-2.57; -0.66)** |  |  |
| Larsson (2020) (19) | European | Yes | log mmol/L per 1-SD increase in log-transformed drinks/week | **-0.37 (-0.67; -0.07)** |  |  |
| Lawlor (2013) (4) | European | Yes | % triglycerides per doubling of alcohol consumption in current drinkers | **-14.9 (-25.6; -4.3)** |  |  |
| Millwood (2019) (16) | Asian | Yes | ln mmol/L per 280 g/week |  | **0.10 (0.04; 0.15)** | (-0.03 (-0.09; 0.02) |
| Peng (2019) (17) | Asian | Yes | log mmol/L for 22 g increase in log-transformed alcohol |  | **0.11 (0.05; 0.16)** | -0.09 (-0.06; 0.23) |
| Silverwood (2014) † (6) | European | Yes | log mmol/L for 1 unit increase in log-transformed alcohol | 0.01 (-0.06; 0.07) |  |  |
| Tabara (2017) (13) | Asian | No | mg/dL per *1 allele vs. *2*2 homozygotes |  | No association |  |
| Taylor (2015) (8) | Asian | No | Times higher log mmol/L per copy of G allele |  | **1.16 (1.09; 1.23)** | 0.98 (0.93; 1.02) |
| Vu (2016) † (11) | European | Yes | ln mg/dL per quartile predicted alcohol consumption (Q3 vs. Q1) | **-0.13 (-0.20; -0.07)** |  |  |
| Zhao (2019) (18) | Asian | Yes | beta coefficient (unit unknown) alcohol vs. no alcohol | 0.08 (-0.20; 0.36) | 0.08 (-0.19; 0.35) | -0.02 (-0.82; 0.77) |
|  |  |  |  |  |  |  |

* Same study population

† These two studies had overlapping study populations

The results presented here are the results of the linear analyses. Detrimental associations have been displayed in **bold red** and beneficial associations in **bold blue**.

Abbreviations: HDL = high-density lipoprotein; IV = instrumental variable analysis; LDL = low-density lipoprotein

**Supplementary Table 5. Overview of the associations of genetically higher alcohol consumption with diabetes-related measures in the included Mendelian randomization studies**

| **Outcome and study** | **Ancestry** | **IV** | **Effect measure and unit** | **Association with outcome** | | |
| --- | --- | --- | --- | --- | --- | --- |
|  |  |  |  | **Total** | **Men** | **Women** |
|  |  |  |  |  |  |  |
| **Fasting glucose** |  |  |  |  |  |  |
| AuYeung (2013) * (2) | Asian | No | mmol/L for GA vs. AA genotype |  | 0.01 (-0.16; 0.14) |  |
| AuYeung (2013) * (3) | Asian | Yes | log mmol/L per 10 g/day |  | 0.01 (-0.006; 0.03) |  |
| Cho (2015) (7) | Asian | Yes | log mg/dL per g/day | **0.0012 (0.0006; 0.0017)** | **0.0010 (0.0006; 0.0014)** | -0.0014 (-0.0052; 0.0025) |
| Jee (2016) (9) | Asian | No | 10 g/day | **1.57 (0.73; 2.41)** | **1.78 (0.97; 2.59)** | -0.21 (-4.17; 3.75) |
| Holmes (2014) (5) | European | No | % difference for GG vs. AA or AG | 0.03 (0.00, 0.06) |  |  |
| Peng (2019) (17) | Asian | Yes | mmol/L per 22 g/week log-transformed alcohol |  | **0.036 (0.018; 0.054)** | 0.014 (-0.036; 0.064) |
| Taylor (2015) (8) | Asian | No | times higher mmol/L per G-allele copy |  | 1.02 (0.99; 1.06) | 0.99 (0.96; 1.01) |
|  |  |  |  |  |  |  |
| **Non-fasting glucose** |  |  |  |  |  |  |
| Lawlor (2013) (4) | European | Yes | % glucose per doubling of alcohol consumption in current drinkers | -2.1 (-5.6, 1.5) |  |  |
| Millwood (2019) (16) | Asian | Yes | mmol/L per 280 g/week |  | **0.15 (0.09; 0.21)** | **-0.07 (-0.12;-0.02)** |
|  |  |  |  |  |  |  |
| **HbA1c** |  |  |  |  |  |  |
| Peng (2019) (17) | Asian | Yes | % per 22 g/week log-transformed alcohol |  | 0.001 (-0.01; 0.013) | -0.004 (-0.037; 0.03) |
|  |  |  |  |  |  |  |
| **2-hour postprandial glucose** |  |  |  |  |  |  |
| Peng (2019) (17) | Asian | Yes | mmol/L per 22 g/week log-transformed alcohol |  | **0.072 (0.035; 0.108)** | **0.064 (0.03; 0.158)** |
|  |  |  |  |  |  |  |
| **HOMA-IR** |  |  |  |  |  |  |
| Peng (2019) (17) | Asian | Yes | mIU × mmol × L^-2^ per 22 g/week log-transformed alcohol |  | **0.104 (0.039; 0.169)** | -0.082 (-0.251; 0.087) |
|  |  |  |  |  |  |  |
| **HOMA-beta** |  |  |  |  |  |  |
| Peng (2019) (17) | Asian | Yes | mIU × mmol × L^-2^ per 22 g/week log-transformed alcohol |  | 0.005 (-0.05; 0.06) | -0.112 (-0.285; 0.062) |
|  |  |  |  |  |  |  |

* Same study population

The results presented here are the results of the linear analyses. Detrimental associations have been displayed in **bold red** and beneficial associations in **bold blue**.

Abbreviations: HbA1c = glycated hemoglobin; HOMA-IR = homeostatic model assessment of insulin resistance; HOMA-beta = homeostatic model assessment of beta-cell function; IV = instrumental variable analysis

**Supplementary References**

1. Chen L, Davey Smith G, Harbord RM, Lewis SJ. Alcohol Intake and Blood Pressure: A Systematic Review Implementing a Mendelian Randomization Approach. PLOS Medicine. 2008;5(3):e52. doi:10.1371/journal.pmed.0050052

2. Au Yeung SL, Jiang C, Cheng KK, et al. Is aldehyde dehydrogenase 2 a credible genetic instrument for alcohol use in Mendelian randomization analysis in Southern Chinese men? Int J Epidemiol. 2013;42(1):318-28. doi:10.1093/ije/dys221

3. Au Yeung SL, Jiang C, Cheng KK, et al. Moderate alcohol use and cardiovascular disease from Mendelian randomization. PLoS One. 2013;8(7):e68054. doi:10.1371/journal.pone.0068054

4. Lawlor DA, Nordestgaard BG, Benn M, Zuccolo L, Tybjaerg-Hansen A, Davey Smith G. Exploring causal associations between alcohol and coronary heart disease risk factors: findings from a Mendelian randomization study in the Copenhagen General Population Study. Eur Heart J. 2013;34(32):2519-28. doi:10.1093/eurheartj/eht081

5. Holmes MV, Dale CE, Zuccolo L, et al. Association between alcohol and cardiovascular disease: Mendelian randomisation analysis based on individual participant data. Bmj. 2014;349:g4164. doi:10.1136/bmj.g4164

6. Silverwood RJ, Holmes MV, Dale CE, et al. Testing for non-linear causal effects using a binary genotype in a Mendelian randomization study: application to alcohol and cardiovascular traits. Int J Epidemiol. 2014;43(6):1781-90. doi:10.1093/ije/dyu187

7. Cho Y, Shin SY, Won S, Relton CL, Davey Smith G, Shin MJ. Alcohol intake and cardiovascular risk factors: A Mendelian randomisation study. Sci Rep. 2015;5:18422. doi:10.1038/srep18422

8. Taylor AE, Lu F, Carslake D, et al. Exploring causal associations of alcohol with cardiovascular and metabolic risk factors in a Chinese population using Mendelian randomization analysis. Sci Rep. 2015;5:14005. doi:10.1038/srep14005

9. Jee YH, Lee SJ, Jung KJ, Jee SH. Alcohol Intake and Serum Glucose Levels from the Perspective of a Mendelian Randomization Design: The KCPS-II Biobank. PLoS One. 2016;11(9):e0162930. doi:10.1371/journal.pone.0162930

10. Tabara Y, Ueshima H, Takashima N, et al. Mendelian randomization analysis in three Japanese populations supports a causal role of alcohol consumption in lowering low-density lipid cholesterol levels and particle numbers. Atherosclerosis. 2016;254:242-8. doi:10.1016/j.atherosclerosis.2016.08.021

11. Vu KN, Ballantyne CM, Hoogeveen RC, et al. Causal Role of Alcohol Consumption in an Improved Lipid Profile: The Atherosclerosis Risk in Communities (ARIC) Study. PLoS One. 2016;11(2):e0148765. doi:10.1371/journal.pone.0148765

12. Almeida OP, McCaul K, Hankey GJ, Yeap BB, Golledge J, Flicker L. Excessive alcohol consumption increases mortality in later life: a genetic analysis of the health in men cohort study. Addict Biol. 2017;22(2):570-8. doi:10.1111/adb.12340

13. Tabara Y, Arai H, Hirao Y, et al. The causal effects of alcohol on lipoprotein subfraction and triglyceride levels using a Mendelian randomization analysis: The Nagahama study. Atherosclerosis. 2017;257:22-8. doi:10.1016/j.atherosclerosis.2016.12.008

14. Cho Y, Kwak S, Lewis SJ, et al. Exploring the utility of alcohol flushing as an instrumental variable for alcohol intake in Koreans. Scientific reports. 2018;8(1):458. doi:10.1038/s41598-017-18856-z

15. Christensen AI, Nordestgaard BG, Tolstrup JS. Alcohol Intake and Risk of Ischemic and Haemorrhagic Stroke: Results from a Mendelian Randomisation Study. J Stroke. 2018;20(2):218-27. doi:10.5853/jos.2017.01466

16. Millwood IY, Walters RG, Mei XW, et al. Conventional and genetic evidence on alcohol and vascular disease aetiology: a prospective study of 500 000 men and women in China. Lancet. 2019;393(10183):1831-42. doi:10.1016/s0140-6736(18)31772-0

17. Peng M, Zhang J, Zeng T, et al. Alcohol consumption and diabetes risk in a Chinese population: a Mendelian randomization analysis. Addiction. 2019;114(3):436-49. doi:10.1111/add.14475

18. Zhao PP, Xu LW, Sun T, et al. Relationship between alcohol use, blood pressure and hypertension: an association study and a Mendelian randomisation study. J Epidemiol Community Health. 2019;73(9):796-801. doi:10.1136/jech-2018-211185

19. Larsson SC, Burgess S, Mason AM, Michaëlsson K. Alcohol Consumption and Cardiovascular Disease: A Mendelian Randomization Study. Circulation. Genomic and precision medicine. 2020;13(3):e002814. doi:10.1161/circgen.119.002814

20. Jiang Q, Wang K, Shi J, Li M, Chen M. No association between alcohol consumption and risk of atrial fibrillation: A two-sample Mendelian randomization study. Nutrition, Metabolism and Cardiovascular Diseases. 2020;30(8):1389-96. doi:<https://doi.org/10.1016/j.numecd.2020.04.014>

21. van Oort S, Beulens JWJ, van Ballegooijen AJ, Handoko ML, Larsson SC. Modifiable lifestyle factors and heart failure: A Mendelian randomization study. American Heart Journal. 2020;227:64-73. doi:<https://doi.org/10.1016/j.ahj.2020.06.007>

22. Yuan S, Larsson SC. An atlas on risk factors for type 2 diabetes: a wide-angled Mendelian randomisation study. Diabetologia. 2020;63(11):2359-71. doi:10.1007/s00125-020-05253-x

23. van Oort S, Beulens JWJ, van Ballegooijen AJ, Burgess S, Larsson SC. Cardiovascular risk factors and lifestyle behaviours in relation to longevity: a Mendelian randomization study. Journal of internal medicine. 2020. doi:10.1111/joim.13196

24. van Oort S, Beulens JWJ, van Ballegooijen AJ, Grobbee DE, Larsson SC. Association of Cardiovascular Risk Factors and Lifestyle Behaviors With Hypertension: A Mendelian Randomization Study. Hypertension (Dallas, Tex. : 1979). 2020;76(6):1971-9. doi:10.1161/hypertensionaha.120.15761
